# CRISPR Ribonucleoprotein-Mediated Precise Editing of Multiple Genes in Porcine Fibroblasts

**DOI:** 10.3390/ani14040650

**Published:** 2024-02-18

**Authors:** Xiaochen Guo, Chang Liu, Yunjing Zhao, Chaoqian Jiang, Junxue Jin, Zhonghua Liu, Yanshuang Mu

**Affiliations:** 1 Key Laboratory of Animal Cellular and Genetic Engineering of Heilongjiang Province, College of Life Science, Northeast Agricultural University, Harbin 150030, China; s190901057@neau.edu.cn (X.G.); s210901071@neau.edu.cn (C.L.); j18845832622@163.com (Y.Z.); jiangchaoqianneau@163.com (C.J.); jinjunxue@neau.edu.cn (J.J.); 2 College of Life Science, Northeast Agricultural University, Harbin 150030, China

**Keywords:** pig, CRISPR/Cas9, precise genome editing, M3814, PS-ssODNs

## Abstract

**Simple Summary:**

The production traits of pigs are generally regulated by multiple candidate genes, which were associated with phenotype through genetic association analysis. In order to study the mechanism of multi-gene regulation during growth and development, it is necessary to establish a multi-gene editing cell model. However, the efficiency of the precise editing of multiple genes is low in pigs. In this study, we established an efficient multi-gene editing system, which produced a significant improvement in knock-in of point mutation efficiency and could edit multiple genes simultaneously. Our results revealed that the optimized multi-gene editing system provides an effective precise multiple gene editing strategy in porcine cells.

**Abstract:**

The multi-gene editing porcine cell model can analyze the genetic mechanisms of multiple genes, which is beneficial for accelerating genetic breeding. However, there has been a lack of an effective strategy to simultaneously perform precise multi-gene editing in porcine cells. In this study, we aimed to improve the efficiency of CRISPR RNP-mediated precise gene editing in porcine cells. CRISPR RNP, including Cas9 protein, sgRNA, and ssODN, was used to generate precise nucleotide substitutions by homology-directed repair (HDR) in porcine fetal fibroblasts (PFFs). These components were introduced into PFFs via electroporation, followed by PCR for each target site. To enhance HDR efficacy, small-molecule M3814 and phosphorothioate-modified ssODN were employed. All target DNA samples were sequenced and analyzed, and the efficiencies of different combinations of the CRISPR RNP system in target sites were compared. The results showed that when 2 μM M3814, a small molecule which inhibits NHEJ-mediated repair by blocking DNA-PKs activity, was used, there was no toxicity to PFFs. The CRISPR RNP-mediated HDR efficiency increased 3.62-fold. The combination of CRISPR RNP with 2 μM M3814 and PS-ssODNs achieved an HDR-mediated precision gene modification efficiency of approximately 42.81% in mutated cells, a 6.38-fold increase compared to the control group. Then, we used the optimized CRISPR RNP system to perform simultaneous editing of two and three loci at the *INS* and *RLN3* genes. The results showed that the CRISPR RNP system could simultaneously edit two and three loci. The efficiency of simultaneous editing of two loci was not significantly different from that of single-gene editing compared to the efficiency of single-locus editing. The efficiency of simultaneous precise editing of *INS*, *RLN3* exon 1, and *RLN3* exon 2 was 0.29%, 0.24%, and 1.05%, respectively. This study demonstrated that a 2 μM M3814 combination with PS-ssODNs improves the efficacy of CRISPR RNP-mediated precise gene editing and allows for precise editing of up to three genes simultaneously in porcine cells.

## 1. Introduction

CRISPR‒Cas9 systems (clustered regularly interspaced short palindromic repeats (CRISPR)–CRISPR-associated protein) have become a simple and common genome editing technique [1,2,3]. In the CRISPR/Cas9 system, Cas9 and sgRNA assemble into a complex that recognizes the targeted DNA sequence and generates target DNA double-strand breaks (DSBs). CRISPR/Cas9-targeted DSBs trigger the DNA repair machinery, which includes two dominant pathways for DSB repair: nonhomologous end joining (NHEJ) and homology-directed repair (HDR). NHEJ often leads to DNA insertions or deletions (indels), while HDR can achieve precise genome editing in the presence of a donor DNA template [1]. Because NHEJ is more efficient than HDR, the CRISPR/Cas9-mediated precise genome modification efficiency is low in several mammalian cells, including pig, mouse, cattle, and human cells [1,2,4,5]. For example, to produce a Huntingtin pig model, 2430 fetal pig fibroblast cells were screened by PCR, and nine positive cell clones were identified [6]. The precise genome modification rate was below 0.5%. This low efficiency limits the applications of CRISPR/Cas9 genome modifications for precise gene editing in pigs [2].

To improve the precise editing efficiency, the components of the CRISPR/Cas9 genome editing system have been modified, including adding small molecules and donor DNA modifications. Many small molecules have shown enhanced CRISPR/Cas9 HDR editing efficiency by inhibiting NHEJ-dependent proteins, such as M3814, which inhibits NHEJ-mediated repair by blocking DNA-PK activity, which is responsible for initiating NHEJ [7,8,9]. M3814 has been proved to enhance the precise genome editing in human cells [10]. The combination of small molecular compounds M3814 and TrichostatinA (TSA) leads to a 3-fold increase in HDR efficiency [11]. In the CRISPR/Cas9 system, it was also reported that ssODNs are more effective donors than traditional double-strand DNA homology for HDR [12]. In addition, ssODNs enable the creation of seamless site-specific mutations and target sites for DNA point mutations or small-fragment insertions [12,13,14]. Two phosphorothioate (phosphate where an oxygen is replaced with a sulfur atom) (PS) linkages at the ends of ssODNs increase the efficiency of gene editing in human cell lines compared to oligos with traditional PO-oxygen bonds [12,15].

Insulin plays an important role in the regulation of glucose utilization in animals. Porcine insulin differs from human insulin by one amino acid (alanine in pigs and threonine in humans) at the carboxy terminus of the B chain (i.e., position B30) [16,17]. Relaxin3 (*RLN3*), a polypeptide hormone that belongs to the insulin superfamily, is involved in a number of functions in mammals, including the stimulation of tissue growth, differentiation, and remodeling during pregnancy [18,19]. Porcine relaxin3 differs from human relaxin3 by three amino acids (alanine in pigs and threonine in humans N25S, S29A, K34R) [18]. The goal of this study was to simultaneously generate precise base replacement in coding region of *INS* and *RLN3* genes in porcine cells.

In this study, we combined two strategies: the administration of a DNA-PKc inhibitor to block the NHEJ pathway and the use of PS-ssODN as a repair template to improve the efficiency of CRISPR RNP-mediated precise genome editing in porcine cells. We demonstrated that PS-ssODN and M3814 produce a significant improvement in the knock-in of point mutation efficiency. The results also showed that this strategy allowed for precise editing of up to three genome sites simultaneously.

## 2. Materials and Methods

### 2.1. Cell Culture

The porcine fetal fibroblasts (PFFs) utilized in this study were sourced from Large White pigs at the Experimental Pig Base of the Embryo Engineering Laboratory, Northeast Agriculture University. All procedures involving animals complied with the guidelines established by the Institutional Animal Care and Use Committees (IACUCs) of Northeast Agriculture University (NO. NEAUEC20190118). The PFF culture method has been previously described [20]. Briefly, PFFs were extracted from a 33-day-old fetus and cultured in Dulbecco’s modified Eagle medium (DMEM, Gibco, New York, NY, USA) supplemented with 15% fetal bovine serum (FBS, HyClone, New York, NY, USA), 1% penicillin‒streptomycin (Gibco, New York, USA), 1% nonessential amino acids (Gibco, New York, NY, USA), and 2 mmol/L L-glutamine (Sigma, New York, NY, USA). The cells were passaged every two days using 0.25% trypsin-EDTA (Gibco, New York, NY, USA).

### 2.2. Design of gRNAs and ssODNs

Two genes, *INS* and *RLN3*, were chosen to design CRISPR targets, with one sgRNA for the *INS* gene and two sgRNAs for the *RLN3* gene. The sgRNA sequences can be found in Supplementary Table S1. To introduce the point mutants, 116 bp ssODNs as donor DNA were introduced into the target using HDR-mediated precise gene editing: one ssODN for the *INS* gene and two ssODNs for the *RLN3* gene. The ssODN sequences are shown in Supplementary Table S2. The sgRNA and ssODN templates with or without thiomodification were synthesized by Genscript (Nanjing, China).

### 2.3. Oligonucleotide and Ribonucleoprotein Electroporation

After trypsin digestion of the PFFs, the digestion was terminated by adding twice the volume of pancreatic enzyme culture medium. The cells were counted, and 1 × 10^6^ cells were taken for use. To prepare the ribonucleoprotein (RNP) mixture, for 1 × 10^6^ cells, 10 μg of Cas9 protein (Genscript, Nanjing, China) was premixed with 100 pmol of sgRNA (Genscript, Nanjing, China) for 10 min at room temperature. Then, 200 pmol of ssODN (Genscript, Nanjing, China) was added and supplemented to a final volume of 20 μL with OptiMEM. The cell pellet containing 1 × 10^6^ cells was resuspended with the RNP mixture, and the mixture was gently pipetted to obtain a uniform cell suspension and was transferred to the electroporation cuvette. The cells were electroporated and transfected according to the established procedure using an electroporation instrument. Following electroporation, the transfected cells were seeded into appropriate petri dishes and continued to culture as needed.

### 2.4. Cell Activity Assay

To evaluate cell activity, the Enhanced Cell Counting Kit-8 (Beyotime, Shanghai, China) was utilized following these steps: Cells were seeded into a 96-well plate at a density of approximately 50%. After 24 h, the culture medium was replaced with M3814-containing medium and incubated for an additional 24 h. Then, 20 μL of the enhanced CCK-8 solution was added to each well and incubated in a cell culture incubator for 0.5–4 h. The absorbance at 450 nm was measured using a microplate reader. The cell viability was calculated using the A450 value obtained.

### 2.5. DNA Extraction and PCR

Genomic DNA was isolated from harvested cells using a Universal Genomic DNA Extraction Kit (Takara, Dalian, Japan) following standard procedures. Subsequently, DNA fragments containing the targeted region, ranging from 200 to 300 bp, were amplified using high-fidelity DNA polymerase. PCR was performed with the following 50 µL reaction: 20 ng genomic DNA, 1 × buffer, 0.5 µM forward primers and reverse primers 0.2 mM dNTP, and 1 U of DNA polymerase (NEB Next Q5, Beijing, China). The PCR conditions were as follows: 98 °C for 5 min, followed by 35 cycles of denaturation at 95 °C for 10 s, annealing at 57 °C for 30 s, and extension at 72 °C for 20 s. The resulting PCR products were analyzed using 1.5% agarose gel electrophoresis. DNA fragments were subsequently purified from the gel using a gel extraction kit (Takara, Dalian, China). The primer sequences can be found in Supplementary Table S3.

### 2.6. Deep Sequencing (Deep-Seq) and Analysis

The PCR amplicons were commercially deep sequenced by Annoroad Gene Technology (Hangzhou, China). Unique barcoded Illumina-compatible adapters were ligated onto each sample during library construction. The libraries were pooled in equimolar concentrations for multiplexed sequencing on the Illumina MiSeq platform using 2 × 150 run parameters. The CRISPResso2 tool was utilized to determine the rates of indels [21].

### 2.7. Statistical Analysis

Statistical analysis was performed using SPSS 16.0 Statistical Software (SPSS, Inc., Chicago, IL, USA). All results are presented as the mean ± standard error (SE) of at least three independent experiments. The *t*-test was employed to compare the observed differences, and statistical significance was defined as *p* < 0.05.

## 3. Results

### 3.1. M3814 Increases CRISPR RNP-Mediated Precise Genome Editing Efficiencies in PFFs

**Figure 1:**
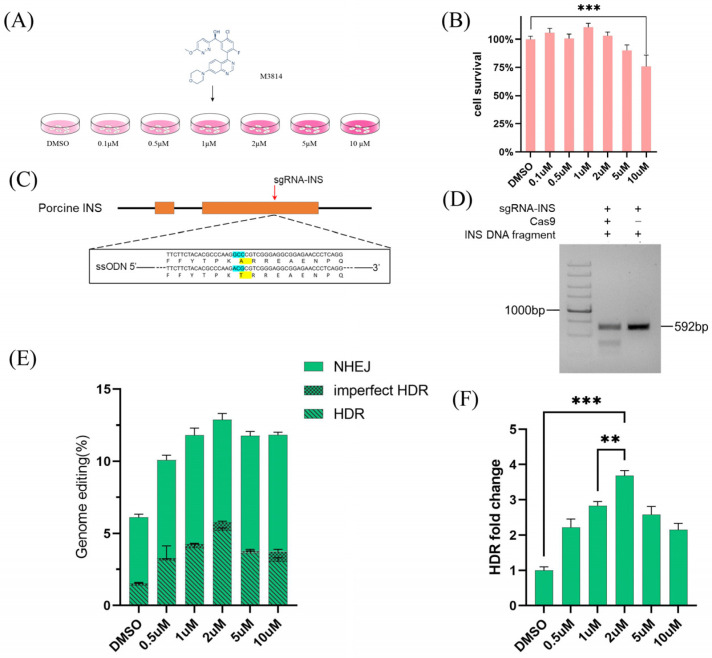
Effects of the gene editing efficiency of CRISPR RNP in PFF by the small-molecule M3814. (**A**) A schematic representation of the CCK8 assay was used to assess cell survival in PFFs after treatment with various concentrations of M3814. (**B**) The CCK8 assay was performed to evaluate cell survival in PFF cells three days after treatment with different concentrations of M3814. (**C**) Schematic of *INS* gene structure and sgRNA-INS target site. (**D**) The cleavage efficiency of sgRNA/Cas9 complexes on PCR-amplified target DNA in vitro was estimated. (**E**) The efficiencies of genome editing for *INS* using Cas9 protein in PFF cells treated with different concentrations of M3814 were determined. (**F**) A histogram illustrating the efficiencies of genome editing for *INS* using Cas9 protein in PFF cells treated with various concentrations of M3814. M3814 was added for 3 days after editing. HDR, homology-directed repair; imperfect HDR, homology-directed repair with indels; NHEJ, nonhomologous end joining; modified, target DNA with altered sequence; unmodified, target DNA without altered sequence. Error bars show the SEM of three replicates for PFFs. *** *p* < 0.001 and ** *p* < 0.01.

To examine the toxic effect of M3814 on PFFs, PFFs were treated with M3814 for 24 h at concentrations of 0, 0.1, 0.5, 1, 2, 5, and 10 μM (Figure 1A). The results showed that treating PFFs with M3814 at 0–2 μM had no effects on cell activity, while treating PFFs with M3814 at 5–10 μM exhibited moderate toxicity (Figure 1B). Previous studies have shown that M3814 increases HDR by inhibiting DNA-PKcs [10]. To determine whether M3418 can improve the efficiency of HDR mediated by CRISPR/Cas9 and ssODN in PFFs, we designed sgRNA and ssODN targeting the *INS* gene in the pig genome (Figure 1C). The cleavage efficiency of sgRNA was confirmed by in vitro assembly of candidate sgRNAs with recombinant Cas9 protein targeting DNA fragments with the target site (Figure 1D). After transfection of PFFs with RNP and ssODN, we treated PFFs with serial concentrations of M3814 (0, 0.5, 1, 2, 5, and 10 μM) for 24 h (Figure 1E and Figure S1). We performed Illumina sequencing of the amplicons around the targeted point mutation sites derived from transfected cell pools (Figure 1E). According to sequence analysis, after treating PFFs with M3814 at 2 μM, 12.87% of the transfected cell pools were mutated cells, and approximately 5.21% of cells exhibited HDR-mediated precise gene modification (Figure 1E and Figure S1). The efficiency of HDR was increased 3.62-fold (Figure 1F). These results showed that M3814 could effectively enhance the efficiency of HDR on PFFs. When 2 μM M3814 was used, the precise gene modification rate was significantly higher than that of the control, and 2 μM M3814 did not cause toxicity to the PFFs.

### 3.2. M3814 in Combination with PS-ssODNs Promotes CRISPR RNP-Mediated Precise Genome Editing Efficiencies in PFFs

**Figure 2:**
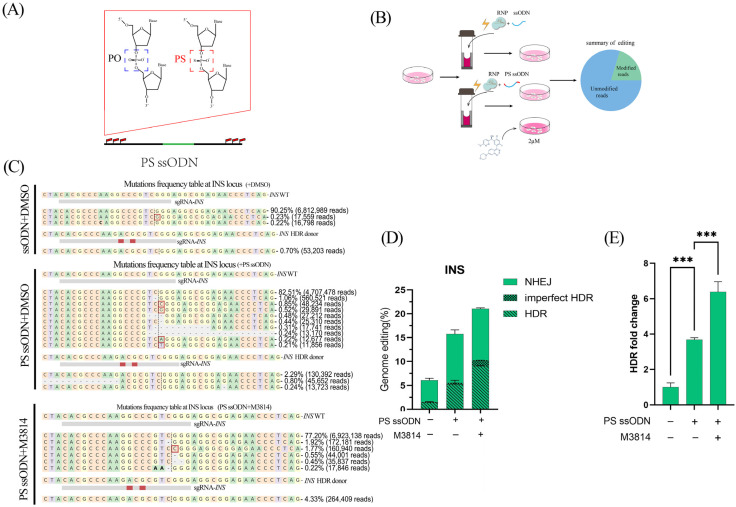
Effects of the gene editing efficiency of CRISPR RNP in PFF by the phosphorothioate-modified ssODN. (**A**) The structure of phosphorothioate-modified ssODN. (**B**) Schematic of the evaluation of the efficiencies of genome editing for *INS* using Cas9 protein in PFF cells with phosphorothioate-modified ssODN and M3814. (**C**) The results of deep sequencing in different groups. (**D**) The efficiencies of genome editing for *INS* using Cas9 protein in PFF cells with phosphorothioate-modified ssODN and M3814. HDR, imperfect HDR (HDR with indels), NHEJ, and unmodified are indicated in yellow, orange, gray and blue, respectively. (**E**) Fold change in the efficiencies of genome editing for *INS* using Cas9 protein in PFF cells with phosphorothioate-modified ssODN and M3814. HDR, homology-directed repair; imperfect HDR, homology-directed repair with indels; NHEJ, nonhomologous end joining; modified, target DNA with altered sequence; unmodified, target DNA without altered sequence. Error bars show the SEM of three replicates for PFF cells. *** *p* < 0.001.

It has been proven that ssODN can be a DNA donor to repair site-specific DSBs under the HDR pathway in mammalian cells [13,14,22]. To test whether ssODN with PS modifications as repair templates can promote insertion repair efficiency in PFFs, we designed 116 nt oligo versions with PS modifications for the porcine *INS* gene A54T point mutations (Figure 2A). The combination of PS-ssODNs with or without 2 μM M3814 was used to examine the precise genome editing efficiency in PFFs (Figure 2B). After CRISPR-mediated gene editing, the efficiencies of precise genome editing were detected by deep sequencing (Figure 2C). These results suggested that when using the PS-modified oligonucleotide, 15.77% of the transfected cell pools were mutated cells, and HDR-mediated precision gene modification was approximately 5.21% of cells. The efficiency of HDR was increased 3.68-fold compared with that of the control (1.41%) (Figure 2D). By using PS-ssODNs combined with 2 μM M3814, HDR-mediated precision gene modification was approximately 9.02% of cells (Figure 2D and Table S1). The efficiency of HDR in the PS-ssODN group with 2 μM M3814 treatment was increased 6.38-fold, higher than that of the control group (*p* < 0.05) (Figure 2E). The efficiency of HDR between the PS-ssODN group with and without 2 μM M3814 was also a significant difference (Figure 2E). By the comparison of the efficiency of HDR between the PS-ssODN group with and without 2 μM M3814, the impact of ssODN on HDR efficiency is greater than that of M3814 on HDR efficiency (Figure 2E).

### 3.3. The Combination of M3814 and PS-ssODNs Achieves Highly Precise Gene Editing Efficiencies at the RLN3 Gene

**Figure 3:**
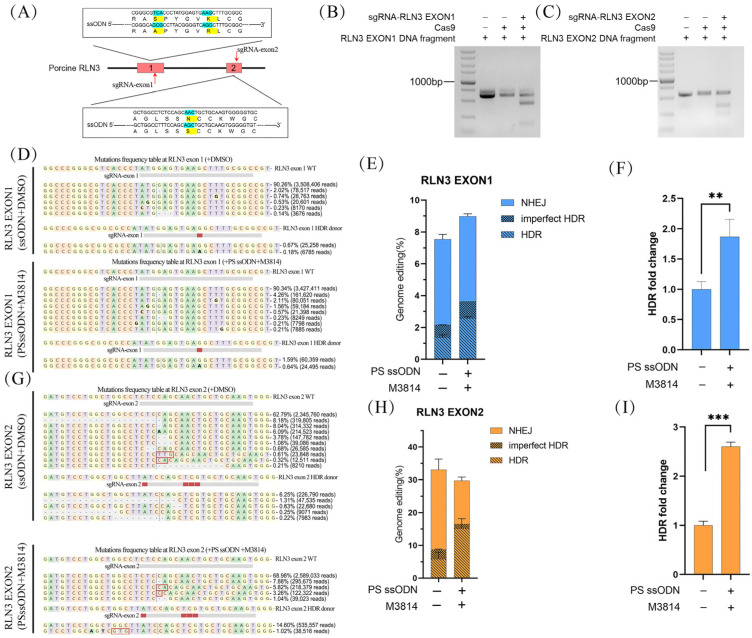
Effects of the gene editing efficiency of CRISPR RNP in PFFs by M3814 and PS-ssODN at the *RLN3* gene. (**A**) Schematic of the *RLN3* gene structure and sgRNA-RLN3 exon 1 and sgRNA-*RLN3* exon 2 target sites. (**B**) Estimating the cleavage efficiency of sgRNA-RLN3 exon 1/Cas9 complexes on PCR-amplified target DNA in vitro. (**C**) Estimating the cleavage efficiency of sgRNA-*RLN3* exon 2/Cas9 complexes on PCR-amplified target DNA in vitro. (**D**) The target regions of *RLN3* exon 1 were aligned with the wild-type sequence using deep sequencing analysis. (**E**) The mutation frequency at the targeting sites of *RLN3* exon 1 in PFFs was examined and calculated using deep sequencing analysis. (**F**) Fold change in genome editing efficiencies of *RLN3* exon 1 with Cas9 protein in PFF cells with PS-ssODN and M3814. Error bars show the SEM of three replicates for PFF cells. (**G**) Deep sequence of *RLN3* exon 2 target regions aligned with that of WT. (**H**) The mutation frequency at the targeting sites of *RLN3* exon 2 in PFFs was examined and calculated using deep sequencing analysis. (**I**) Fold change in genome editing efficiencies of *RLN3* exon 2 with Cas9 protein in PFF cells with PS-ssODN and M3814. Error bars show the SEM of three replicates for PFF cells. M3814 was added for 3 days after editing. HDR, homology-directed repair; imperfect HDR, homology-directed repair with indels; NHEJ, nonhomologous end joining; modified, target DNA with altered sequence; unmodified, target DNA without altered sequence. Error bars show the SEM of three replicates for PFF cells. *** *p* < 0.001 and ** *p* < 0.01.

To verify whether this strategy can achieve high efficiency at other gene loci, we designed two sgRNAs targeted to pig *RLN3* gene exon 1 and exon 2. sgRNA-RLN3 exon 1 with PS-ssODN-RLN3 exon 1 was used to generate S29A and K34R amino acid mutations, and sgRNA-RLN3 exon 2 with PS-ssODN-RLN3 exon 2 was used to generate N25S amino acid mutations in the pig *RLN3* gene (Figure 3A). The cleavage efficiency of sgRNA-RLN3 exon 1 and sgRNA-RLN3 exon 2 was confirmed through in vitro assembly of recombinant Cas9 protein with candidate sgRNAs, targeting DNA fragments with the specific target site (Figure 3B,C). After CRISPR-mediated gene editing, the efficiencies of precise genome editing were detected by deep sequencing (Figure 3D,G). These results suggested that HDR-mediated precision gene modification of *RLN3* exon 1 occurred in approximately 2.57% of cells (Figure 3E and Table S2). The proportion of precision-edited cells to total cells was increased 1.87-fold compared to the control group (Figure 3F). HDR-mediated precision gene modification of *RLN3* exon 2 was observed in approximately 15.14% of cells (Figure 3H and Table S3). The proportion of precision-edited cells to total cells was increased 2.61-fold compared to the control group (Figure 3I).

### 3.4. Simultaneous Precise Editing of Multiple Genes Mediated by CRISPR RNP in PFFs

**Figure 4:**
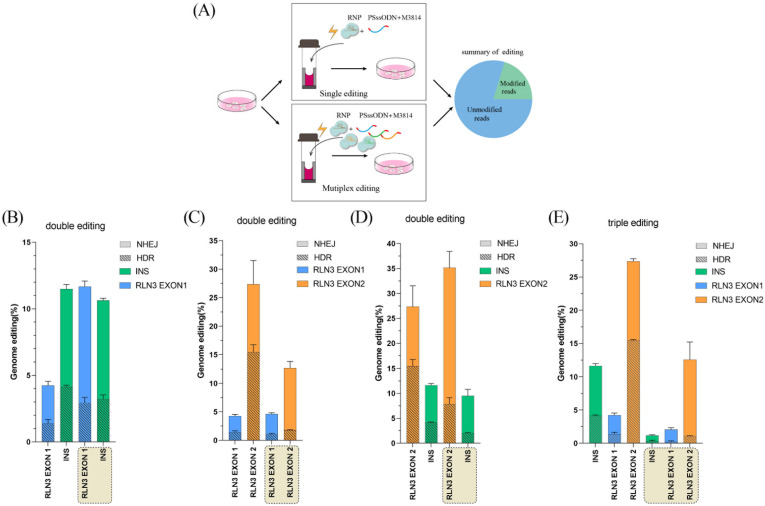
Multiplexed precise genome editing in PFF by CRISPR RNP, M3814 and phosphorothioate-modified ssODN. (**A**) Schematic of the evaluation of the genome editing efficiencies of single editing and multiplex editing with Cas9 protein in PFF cells with PS-ssODN and M3814. (**B**) The gene editing efficiencies of *RLN3* exon 1 and *INS* after simultaneous gene editing with Cas9 protein in PFF cells treated with 2 μM M3814 and phosphorothioate-modified ssODN. (**C**) The gene editing efficiencies of *RLN3* exon 1 and *RLN3* exon 2 after simultaneous gene editing with Cas9 protein in PFF cells treated with 2 μM M3814 and phosphorothioate-modified ssODN. (**D**) The gene editing efficiencies of *RLN3* exon 2 and *INS* after simultaneous gene editing with Cas9 protein in PFF cells treated with 2 μM M3814 and phosphorothioate-modified ssODN. (**E**) The gene editing efficiencies of *RLN3* exon 1, *RLN3* exon 2 and *INS* after simultaneous gene editing with Cas9 protein in PFF cells treated with 2 μM M3814 and PS-ssODN. M3814 was added for 3 days after editing. HDR, homology-directed repair; NHEJ, nonhomologous end joining. Error bars show the SEM of three replicates for PFF cells.

To expand the application scope of this precise editing strategy, we performed simultaneous editing of two loci or three loci simultaneously in PFFs (Figure 4A). After CRISPR-mediated gene editing, the efficiencies of precise genome editing were detected by deep sequencing (Figure S1). The efficiency of simultaneous precise editing at the *INS* and *RLN3* exon 1 loci was 3.21% and 2.92%, respectively (Figure 4B). The efficiency of simultaneous editing of two loci was not significantly different from that of single gene editing compared to the efficiency of single-locus editing (Figure 4B). The efficiency of simultaneous precise editing at the *RLN3* exon 1 and *RLN3* exon 2 loci was 1.79% and 1.04%, respectively (Figure 4C). The efficiency of simultaneous editing of two loci was not significantly different from that of single gene editing compared to the efficiency of single-locus editing (Figure 4C). The efficiency of simultaneous precise editing at the *INS* and *RLN3* exon 2 loci was 1.99% and 7.83%, respectively (Figure 4D). The efficiency of simultaneous editing of two loci was not significantly different from that of single gene editing compared to the efficiency of single-locus editing (Figure 4D). The efficiency of simultaneous precise editing of *INS*, *RLN3* exon 1, and *RLN3* exon 2 was 0.29%, 0.24%, and 1.05%, respectively (Figure 4E). The efficiency of HDR-mediated precise gene modification was significantly lower when three loci were edited simultaneously compared to the efficiency of precise editing of individual genes (Figure 4E).

## 4. Discussion

The CRISPR/Cas9 system is a groundbreaking genome editing technology, renowned for its high efficiency in modifying target genes within mammalian cells [23]. In pigs, previous studies have shown that CRISPR/Cas9 has been used in gene function research and genetic breeding [2,4,24]. CRISPR/Cas9 has precisely genetically modified the genome in pig somatic cells and generated pig models by somatic cell nuclear transfer (SCNT) for translational biomedical research [2,4]. This is especially pertinent to livestock species cell lines, which are historically extremely difficult to modify because of embryonic stem cell shortages [25]. However, the precise genome editing efficiency mediated by CRISPR/Cas9 is still low in livestock species somatic cells [26]. With the development of gene editing technologies, the issue of improving precise genome editing efficiency has become a crucial topic in this field.

As precise genome editing mediated by CRISPR/Cas9 relies on HDR, several strategies have been developed to increase HDR in mammalian cells and mouse zygotes by inhibiting the NHEJ pathway [27,28,29]. The NHEJ pathway is the primary repair mechanism in mammalian cells, as compared to HDR pathways [30]. Thus, inhibiting the function of key proteins involved in the NHEJ pathway may provide a potential approach for augmenting the efficacy of HDR and enhancing precise genome editing facilitated by CRISPR/Cas9 [15,31]. The kinase function of DNA-PKcs plays a crucial role in initiating the NHEJ process. A reduction in the expression of DNA-PKcs resulted in a 2.8-fold increase in HDR events and a 3-fold decrease in NHEJ events [32]. Moreover, the efficiency of homology-directed repair (HDR) induced by double-strand breaks (DSBs) was increased two to three-times in cells with catalytically inactivated DNA-PKcs when a lysine to arginine mutation was introduced at this site, in comparison to DNA-PKcs^–/–^ cells [33]. The use of M3814, a powerful and selective inhibitor of DNA-PKcs, resulted in a significant boost in the frequency of CRISPR/Cas9-mediated HDR, elevating it from 18% to 81% in K562 cells, demonstrating moderate toxicity [34]. A similar enhancing effect of M3814 was observed in hiPSCs [21]. Our results showed that 2 µM M3814 increased HDR events by 3.68-fold in pig PFFs. Therefore, inhibiting the activity of DNA-PKcs enhances the competing activity of HDR and, thereby, enhances the efficiency of CRISPR/Cas9-mediated HDR in porcine.

Numerous studies have demonstrated enhanced efficiency in CRISPR/Cas9 genome editing through modifications of its components, particularly donor DNA [35,36]. The use of single-stranded oligodeoxynucleotide (ssODN) as the donor template has been reported to enhance the rates of HDR by lowering the risk of integration at the cleavage site within the genome [37]. Single-stranded DNA (ssDNA) yields higher rates of targeted knock-in (KI) compared to double-stranded DNA (dsDNA). Unlike dsDNA, ssDNA is unable to randomly integrate into the genome [38]. An effective and affordable method to enhance the efficiency of precise gene modification is by introducing phosphorothioate (PS) linkages at the ends of the oligo, effectively blocking the activity of exonucleases [37]. In this study, the results showed that PS-ssDNA had higher targeted KI rates than ssDNA. The efficiency of HDR was increased by 3.68-fold compared with that of the control.

In fact, the efficiency of precise genome editing can potentially be further enhanced by combining single-stranded DNA (ssDNA) with other approaches that augment precise genome editing, such as the incorporation of small molecules [12,39,40,41]. It was reported that the repair of Cas9/sgRNA-induced DSBs via HDR was markedly increased by a combination of small molecules and single-stranded oligodeoxynucleotides (ssODNs) [42]. Activating HDR-dependent protein, RAD51, and RS-1 significantly enhances the efficiency of CRISPR-mediated targeted knock-in in bovine embryos [41]. The combination of the co-expression CRISPR/Cas9 vector, ssODN, and ligase IV inhibitor SCR7 can markedly improve CRISPR/Cas9-directed gene editing in human cancer cells [14]. In the present study, the results showed that the efficiency of HDR by the combination of M3814 with PS-ssODNs HDR-mediated precision gene modification was increased 6.38-fold compared with that of the control group. These studies made great contributions to the application in targeted gene editing of CRISPR/Cas9 system.

In this study, insulin has a site that needs to be modified [42,43]. *RLN3* has three sites that need to be modified, of which two are closer and can be modified in a donor template [18]. To improve efficiency and save operational processes, simultaneous precise genome editing of multiple genes was carried out. Regardless of whether both genes were located on different chromosomes, different chromosome arms, or the same chromosome arm, the frequency of HDR for double edits was comparable or slightly lower than that of single edits for the respective genes. The average HDR efficiency for the combinations of three gene loci was one-third lower compared to the efficiency of single edits. Overall, the approach established in this study enables the simultaneous editing of multiple target genes in pig cells. This method can also be used to create cell models of related point mutations for exploring the mechanism of single-nucleotide polymorphism (SNP) loci, which are significantly associated with production traits in genetic breeding.

## 5. Conclusions

In conclusion, M3814 is a successful strategy to enhance HDR by CRISPR RNP-mediated DSB in pig fetal fibroblasts. The PS-ssODN donor was more efficient in introducing gene knock-in than using ssODN in the CRISPR RNP system. The efficiency of CRISPR RNP-mediated precise genome editing was improved by the combination of M3814 with PS-ssODN donors, providing a reliable strategy for precise gene editing of multiple genes simultaneously in pigs. The results of this study provide an efficient tool for precise genome editing in domesticated animals.

## Data Availability

The authors declare that all data supporting the findings of this study are available within the paper and its Supplementary Materials.

## Supplementary Materials

The following supporting information can be downloaded at: https://www.mdpi.com/article/10.3390/ani14040650/s1. Figure S1: The sequence results of double and triple editing with Cas9 protein in PFF cells treated with 2 μM M3814 and phosphorothioate-modification ssODN; Table S1: sgRNA Target Sites; Table S2: ssODN synthesized in this paper; Table S3: Primer Sequence for PCR.
