# Supplementary material for: CRISPR Ribonucleoprotein-Mediated Precise Editing of Multiple Genes in Porcine Fibroblasts
Source: Animals (Basel). 2024 Feb 18;14(4):650. doi: 10.3390/ani14040650 (PMC10886166; doi:10.3390/ani14040650)
Supplement: Supplementary file 1 [file animals-14-00650-s001.zip › Supplementary Figure 1.pdf]

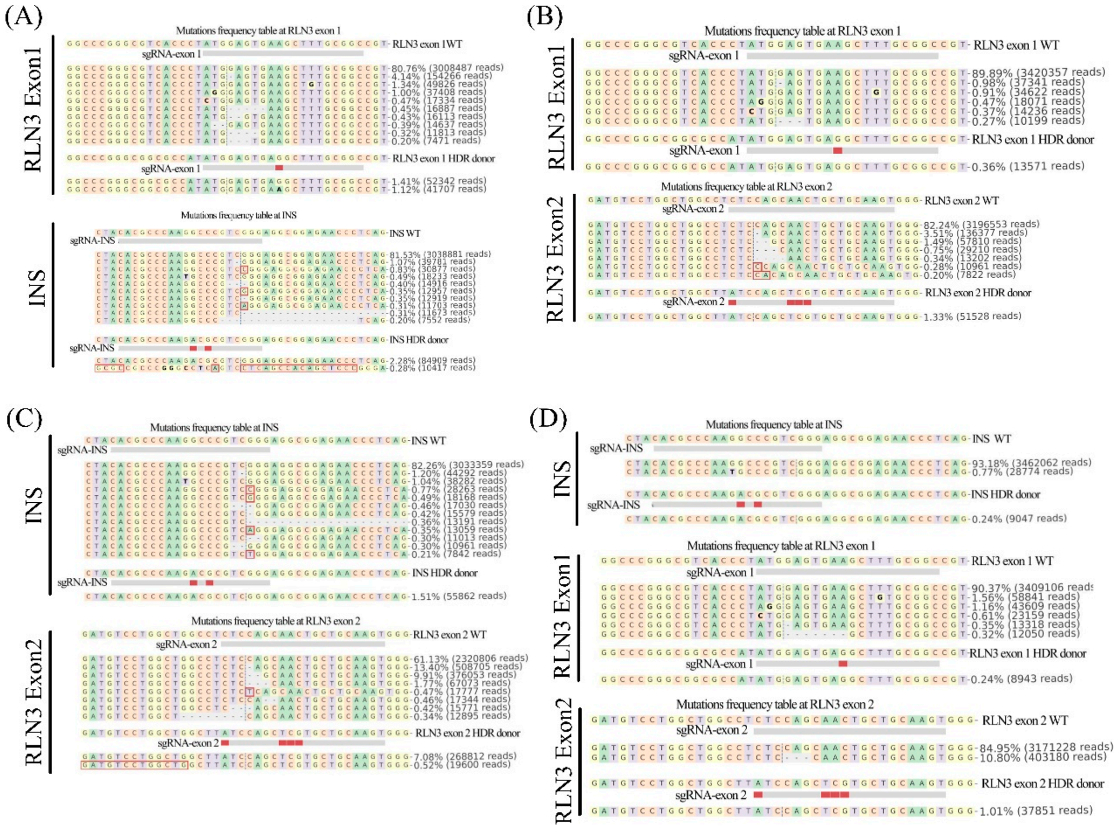

Supplementary Figure 1. The sequence results of double and triple editing with Cas9 protein in PFF cells treated with 2 μM M3814 and phosphorothioate-modification ssODN. (A)The gene editing results of RLN3 Exon1 and INS after simultaneously gene editing with Cas9 protein in PFF cells treated with 2 μM M3814 and phosphorothioate-modification ssODN. (B) The gene editing results of RLN3 Exon1 and RLN3 Exon2 after simultaneously gene editing with Cas9 protein in PFF cells treated with 2 μM M3814 and phosphorothioate-modification ssODN. (C) The gene editing results of RLN3 Exon2 and INS after simultaneously gene editing with Cas9 protein in PFF cells treated with 2 μM M3814 and phosphorothioate-modification ssODN. (D) The gene editing results of RLN3 Exon1, RLN3 Exon2 and INS after simultaneously gene editing with Cas9 protein in PFF cells treated with 2 μM M3814 and phosphorothioate-modification ssODN.
