# Supplementary material for: CRISPR Ribonucleoprotein-Mediated Precise Editing of Multiple Genes in Porcine Fibroblasts
Source: Animals (Basel). 2024 Feb 18;14(4):650. doi: 10.3390/ani14040650 (PMC10886166; doi:10.3390/ani14040650)
Supplement: Supplementary file 1 [file animals-14-00650-s001.zip › Supplementary Table 1. sgRNA Target Sites.pdf]

Supplementary Table 1. sgRNA Target Sites

Gene Name	sgRNA sequence (5'-3')	PAM Sequence
INS	CACGCCCAAGGCCCGTCGGG	AGG
RLN3 Exon1	GCCGCAAAGCTTCACTCCAT	AGG
RLN3 Exon2	ACTTGCAGCAGTTGCTGGAG	AGG
