# Supplementary material for: CRISPR Ribonucleoprotein-Mediated Precise Editing of Multiple Genes in Porcine Fibroblasts
Source: Animals (Basel). 2024 Feb 18;14(4):650. doi: 10.3390/ani14040650 (PMC10886166; doi:10.3390/ani14040650)
Supplement: Supplementary file 1 [file animals-14-00650-s001.zip › Supplementary Table 2. ssODN synthesized in this paper.pdf]

Supplementary Table 2. ssODN synthesized in this paper

Gene (Architecture)	Sequence(5'->3')
INS	ACGCCCCCTCGGCTCACCCTGAGGGTTCTCCGCCTCCCGACGCGTCTTGGGCGTGTAGAAGAAGCCGCGCTCCCCGCACACCAGGTACAGCGCCTCCACCAGGTGGGAGCCGCACA
RLN3 Exon1-	TGCTGGCTGGGGAGCTGTGGCTGAGGACTGAGGCCCGGGCGGCGCCATATGGAGTGAGGCTTTGCGGCCGTGAATTCATCCGAGCGGTCATCTTTACCTGCGGGGGCTCCCGGTGGAGA
RLN3 Exon2	TGGGGCTCTTAGGGGCAGTCGTGATGTCCTGGCTGGCTTATCCAGCTCGTGCTGCAAGTGGGGGTGCAGCAAGAGTGAAATCAGCAGCCTCTGCTAGACAGGGGACCAGGTGGCCATGGAGAA
