# Supplementary material for: CRISPR Ribonucleoprotein-Mediated Precise Editing of Multiple Genes in Porcine Fibroblasts
Source: Animals (Basel). 2024 Feb 18;14(4):650. doi: 10.3390/ani14040650 (PMC10886166; doi:10.3390/ani14040650)
Supplement: Supplementary file 1 [file animals-14-00650-s001.zip › Supplementary Table 3. Primer Sequence for PCR.pdf]

Supplementary Table 3. Primer Sequence for PCR

Primer Name	Primer sequence(5'-3')	Gene	Product
RLN3-E1F	CAACCATCAGAAAGCTGCCTTG	RLN3 Exon1	607bp
RLN3-E1R	CATTACCTCCCCAGCCTCACC		
RLN3-E2F	GCAATGGCCTCCAGCGAAT	RLN3 Exon1	553bp
RLN3-E2R	GCCCACCTGGACACCCAT		
INS-F	CCGGCCCAGGCCTTCGTGAACCA	INS Exon	592bp
INS-R	GGCTTCTCGAGCGGGACCGGG		
